# *Lactobacillus frumenti* mediates energy production via fatty acid β-oxidation in the liver of early-weaned piglets

**DOI:** 10.1186/s40104-019-0399-5

**Published:** 2019-12-05

**Authors:** Zhichang Wang, Jun Hu, Wenyong Zheng, Tao Yang, Xinkai Wang, Chunlin Xie, Xianghua Yan

**Affiliations:** 1 State Key Laboratory of Agricultural Microbiology, College of Animal Sciences and Technology, Huazhong Agricultural University, Wuhan, Hubei 430070 China; 2 The Cooperative Innovation Center for Sustainable Pig Production, Wuhan, Hubei 430070 China; 3 Hubei Provincial Engineering Laboratory for Pig Precision Feeding and Feed Safety Technology, Wuhan, Hubei 430070 China

**Keywords:** Early-weaned piglets, Fatty acid β-oxidation, Gut microbiota, *Lactobacillus frumenti*, Metabolomic analysis

## Abstract

**Background:**

Early-weaning of piglets is often accompanied by severe disorders, especially diarrhea. The gut microbiota and its metabolites play a critical role in the maintenance of the physiologic and metabolic homeostasis of the host. Our previous studies have demonstrated that oral administration of *Lactobacillus frumenti* improves epithelial barrier functions and confers diarrhea resistance in early-weaned piglets. However, the metabolic response to *L. frumenti* administration remains unclear. Then, we conducted simultaneous serum and hepatic metabolomic analyses in early-weaned piglets administered by *L. frumenti* or phosphate-buffered saline (PBS).

**Results:**

A total of 100 6-day-old crossbred piglets (Landrace × Yorkshire) were randomly divided into two groups and piglets received PBS (sterile, 2 mL) or *L. frumenti* (suspension in PBS, 10^8^ CFU/mL, 2 mL) by oral administration once per day from 6 to 20 days of age. Piglets were weaned at 21 days of age. Serum and liver samples for metabolomic analyses were collected at 26 days of age. Principal components analysis (PCA) showed that *L. frumenti* altered metabolism in serum and liver. Numerous correlations (*P* < 0.05) were identified among the serum and liver metabolites that were affected by *L. frumenti*. Concentrations of guanosine monophosphate (GMP), inosine monophosphate (IMP), and uric acid were higher in serum of *L. frumenti* administration piglets. Pathway analysis indicated that *L. frumenti* regulated fatty acid and amino acid metabolism in serum and liver. Concentrations of fatty acid β-oxidation related metabolites in serum (such as 3-hydroxybutyrylcarnitine, C4-OH) and liver (such as acetylcarnitine) were increased after *L. frumenti* administration.

**Conclusions:**

Our findings suggest that *L. frumenti* regulates lipid metabolism and amino acid metabolism in the liver of early-weaned piglets, where it promotes fatty acid β-oxidation and energy production. High serum concentrations of nucleotide intermediates, which may be an alternative strategy to reduce the incidence of diarrhea in early-weaned piglets, were further detected. These findings broaden our understanding of the relationships between the gut microbiota and nutrient metabolism in the early-weaned piglets.

## Background

In the modern intensive swine industry, early weaning strategy can enhance the whole growth performance, but this process also involves complex psychologic, immunologic, environmental, and nutritional stresses [1, 2]. Diarrhea is a major challenge for early-weaned piglets which led to gut microbial dysbiosis, intestinal damage and inflammation, body weight loss, and potentially death [1–3]. The antibiotics overuse and the consequent drug resistance raises serious concerns about both animal and public health. To prevent this problem worsening, the identification or development of alternatives to the established antibiotics for use in early-weaned pigs represents an urgent challenge [2].

Non-antimicrobial alternatives, such as probiotics [4], prebiotics [5], and essential oils [6], have the ability to diminish weaning-associated challenges, especially post-weaning diarrhea. Importantly, probiotics confer a health benefit to the host. Probiotics have been shown to be effective in growth promotion, high feed utilization efficiency, improvement of gastrointestinal conditions, activation of the immune system, restoration of gut microbial balance, and treatment of gastrointestinal disorders [7]. These effects imply that probiotics may be useful in the management of the weaning phase in pigs as alternatives to antibiotics.

The effects of probiotics in weaned piglets have been widely documented, such as protection of piglets from post-weaning infections, maintenance of intestinal epithelial barrier function and stimulation of the piglets immune system [1]. Lactic acid-producing bacteria [2], such as *Lactobacillus*, *Bifidobacterium*, *Bacillus*, *Enterococcus*, or *Streptococcus* and *Saccharomyces*, are the most commonly used microorganisms. Several recent studies have shown that the administration of *Lactobacillus* and *Bifidobacterium* spp. restores the gut microbiota and health [8–11]. Despite the fact that the underlying mechanisms of these effects are not well understood, the prevention of post-weaning disorders in piglets using probiotics could be the result of the inhibition of pathogen adhesion to the intestinal mucosa and their growth, improvement of intestinal epithelial barrier functions, or modifications in the diversity or composition of the gut microbiota.

Our previous study has found that *L. frumenti* significantly increased the body weights [12] and significantly decreased the diarrhea incidence of the early-weaned piglets [13]. These studies have shown that *L. frumenti* improves the intestinal epithelial barrier function and confers diarrhea resistance to early-weaned piglets. However, the effects of *L. frumenti* administration on whole-body metabolism have not been well-characterized. Therefore, the purpose of this study was to determine the effects of *L. frumenti* on the serum and liver metabolomic profiles of early-weaned piglets. To this end, metabolomics, using a mass spectrometry-based technique, was employed to identify the metabolic differences in early-weaned piglets administered with *L. frumenti* or PBS in this study.

## Methods

### Animals and sample collection

The bacterial strain *Lactobacillus frumenti* was isolated from the feces of piglets, as previously described [13]. All animal experimental procedures (permit number: HZAUSW2013–0006) were performed using protocols approved by the Institutional Animal Care and Use Committee of Huazhong Agricultural University. A total of 100 crossbred piglets (Landrace × Yorkshire) of similar birth weight from 15 litters were randomly allocated to two groups (group A, PBS and group B, *L. frumenti*). Each group was then randomly assigned to five pens, which pen size is one sow with a litter size of 10 (five males and five females). At 6 days of age, male piglets were castrated. Piglets in group A were treated daily with 2 mL sterile PBS by oral gavage from age of 6–20 days. Piglets in group B were treated daily with 2 mL L. frumenti suspension in PBS (10^8^CFU/mL) by oral administration from age of 6–20 days. The piglets were then weaned at 21 days of age. All the piglets had free access to diet and water. The diet compositions for early-weaned piglets was as previously described [12]. At 26 days of age, one male piglet was randomly selected from each pen and slaughtered 12 h after the last meal. The blood sample was collected and serum was separated by centrifugation at 1500×*g* for 10 min at 4 °C. The liver sample was washed twice with PBS and then rapidly frozen in liquid nitrogen. The serum and liver samples were stored at − 80 °C until further analysis.

### Metabolomic profiling

Liver samples were ground into a fine powder in liquid nitrogen and serum samples were thawed on ice before metabolite extraction. Firstly, 100 mg liver powder or 100 μL serum sample were mixed with 400 μL methanol for 60 min at − 20 °C to precipitate proteins, and the extracts were harvested by centrifugation at 14,000×*g* for 15 min at 4 °C. The supernatants were transferred to new vials, and aliquots were taken and dried under nitrogen and then under vacuum overnight. Aliquots from each sample were reconstituted in solvent mixtures and shaken for 5 min, prior to centrifugation at 14,000×*g* for 15 min at 4 °C. Prior to liquid chromatography (LC)-mass spectrometry (MS)/MS analysis, an aliquot from each sample was pooled to create quality control (QC) samples that were used to evaluate the internal standards and instrument performance.

The samples were analyzed using a Thermo Q Exactive HF Hybrid Quadrupole-Orbitrap Mass Spectrometer connected to a Thermo Dionex UltiMate 3000 HPLC system (Thermo Scientific, USA). The UltiMate 3000 HPLC system was equipped with a hydrophobic interaction liquid chromatography (HILIC) column. The HILIC column was a Thermo Accucore HILIC column (100 × 3 mm, internal diameter 2.6 μm, part number: 17526-10330). The column was warmed to 40 °C before use. The HILIC column was used as follows: mobile phase A was 10 mmol/L ammonium acetate and mobile phase B was acetonitrile. The gradient was: 0–1 min, 5% B; 1–2 min, a linear gradient from 5% B to 40% B; 2–11 min, a linear gradient from 40% B to 80% B; 11–15 min, 95% B. The electrospray ionization source on the Q Exactive HF Hybrid Quadrupole-Orbitrap Mass Spectrometer was set as follows: the spray voltage was set to 3.8 kV in ES+ and 3.2 kV in ES−; the flow rates of the sheath gas and aux gas were 35 arb and 10 arb, respectively; and the capillary temperature was 350 °C.

### Validation of the important metabolites

Nucleotides and fatty acid oxidation metabolites in this manuscript were confirmed by reverse-phase high-performance liquid chromatography [14] and amino acids and *DL*-lactic acid were confirmed as described previously [15].

### Data processing

An untargeted metabolomic workflow with identification using mzCloud (ddMS2), ChemSpider (formula or exact mass), and Kyoto Encyclopedia of Genes and Genomes (KEGG) pathways was used for the processing of the raw data. All the raw files generated in the QE HF-X analysis were processed using Compound Discoverer 2.0 software (Thermo Scientific, San Jose, CA, USA). Blank injections were used to remove background ion peaks. Quantification data were normalized to the output from QC samples, and the metabolites that were present at significantly different concentrations between the groups were identified using a variable influence on projection (VIP) of > 1 and a *P* value of *t*-tests statistics < 0.05 based on the peak intensities.

### Correlation analysis

To identify significant temporal correlations among metabolites in each tissue, we applied a robust permutation test, which performed 1000 random permutations of the replicate samples, and estimated the Pearson correlation coefficients and significance levels. Correlations between metabolites with *P* values < 0.05 in 95% (99% within tissue Circos plots) of all permutation tests were considered to be significant. Calculations were performed using the R package 3.5.1.

### Western blotting

Liver samples were lysed using lysis buffer (50 mmol/L Tris, 150 mmol/L NaCl, 1 mmol/L EDTA, 1% Triton X-100) supplemented with protease inhibitors (1 μg/mL leupeptin, 1 μg/mL aprotinin, 1 μg/mL pepstatin, and 50 μg/mL polymethyl sulfonyl fluoride). Equal amounts of tissue lysate were resolved using 10% sodium dodecyl sulfate-polyacrylamide gel electrophoresis. The proteins were then transferred onto polyvinylidene fluoride membranes (16916600, Roche), blocked with 5% non-fat milk in Tris-buffered saline containing 0.1% Tween 20 for 1 h at room temperature, and probed with primary antibodies against acyl-coenzyme A oxidase 1 (ACOX1) (AB091), long-chain fatty acid CoA ligase 1 (ACSL1) (A1000), short-chain acyl-CoA dehydrogenase (ACADS) (A0945), carnitine palmitoyl transferase 1 (CPT1) (A5307), and β-actin (A026) (all from ABclonal Technology). The specific proteins were detected using horseradish peroxidase-conjugated secondary antibodies (sc-2004, Santa Cruz Biotechnology), developed with SuperSignal West Pico Chemiluminescent Substrate (34,080, Thermo Scientific), and visualized using a Kodak Image Station 2000 MM. Immunoblotting results were quantified using Image J software (1.49 s). All the immunoblotting assays were performed using three biologic replicates.

### Statistical analysis

Statistical analysis of the western blotting data was performed using GraphPad Prism software (6.0c). All values are shown as mean ± standard deviation (SD), and statistical significance was calculated using two-tailed Student’s *t*-tests. Differences were considered statistically significant at *P* < 0.05.

## Results

### Metabolomic profiling of serum and liver from early-weaned piglets orally administered *L. frumenti*

**Fig. 1:**
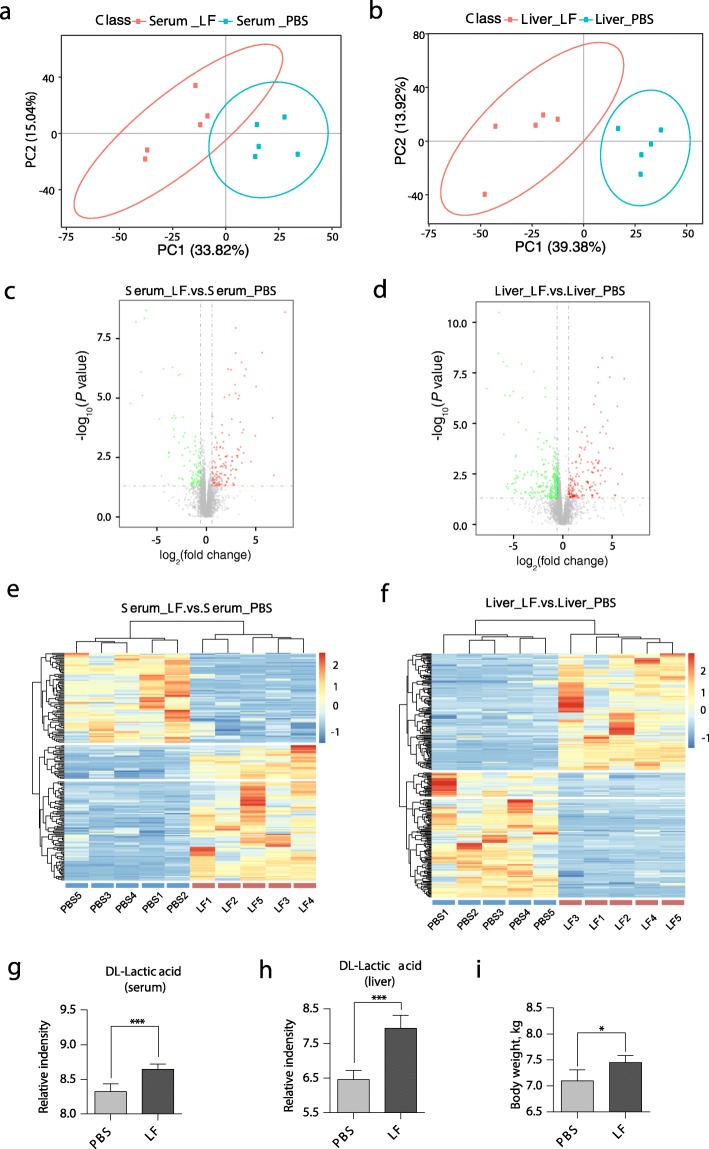
Serum and liver metabolic profiles of piglets orally treated with *L. frumenti* or PBS. **a**, **b** Principal components analysis of serum and liver metabolites in early-weaned piglets gavaged with PBS or *L. frumenti*. **c**, **d** Volcano plot summarizing the distribution of the metabolites. Red, green, and gray dots represent metabolites with higher, lower, or similar abundance, respectively. **e**, **f** Heat map of the metabolites present at differing concentrations in serum and liver of the treated and control early-weaned piglets. **g**, **h** Representative variation of *L. frumenti* metabolism in serum and liver. LF, *L. frumenti*. **i** The body weights of early-weaned piglets used in metabolomic study. All values are shown as mean ± standard deviation (SD), and statistical significance was calculated using two-tailed Student’s *t*-tests. Differences were considered statistically significant at *P* < 0.05 (*) and *P* < 0.001 (***). *n* = 5 piglets

The PBS and *L. frumenti*-treated groups could be readily differentiated using their metabolite fingerprints, with excellent separation of the PBS and *L. frumenti-*treated early-weaned piglets being achieved using the first two components of a principal component analysis (PCA) (Fig.1a and b). The metabolomic analysis permitted the identification and quantification of 2074 metabolites in both serum and liver (Fig. 1c and d), of which 100 were present at concentrations that differed between the groups in the serum and 113 in the liver (fold change > 1.5, or fold change < 0.67). These profiles are displayed as heat maps (Fig. 1e and f). piglets. *DL*-Lactic acid, a product of fermentation by *Lactobacilli,* is upregulated in both serum and liver (Fig. 1g and h). And the body weights of early-weaned piglets were significantly increased by oral administration of *L. frumenti* (Fig. 1i). These data indicate that oral administration of *L. frumenti* significantly modulates the metabolism of early-weaned piglets.

### Correlations in metabolite concentrations within tissues reveal specific differences in metabolism

**Fig. 2:**
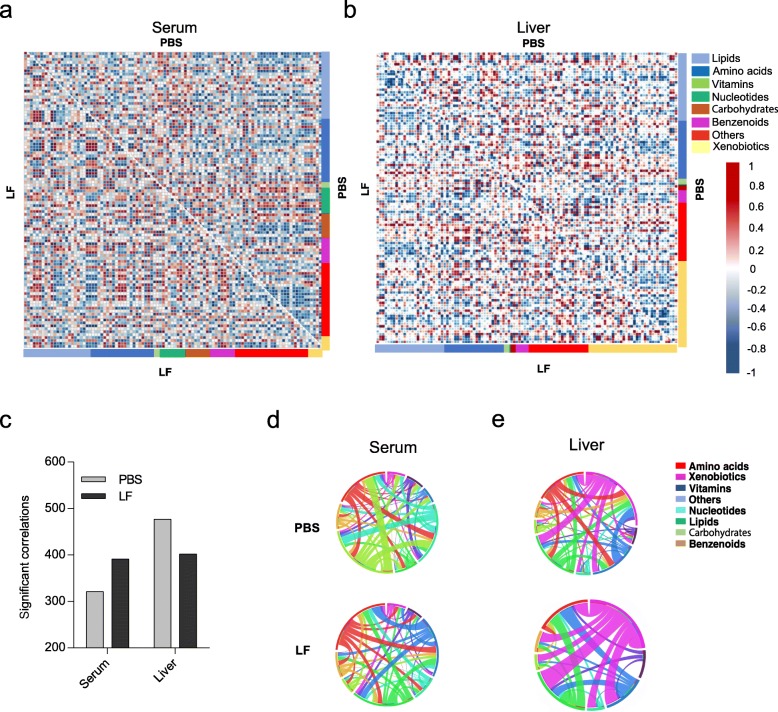
Correlations between serum and liver metabolites that were altered by *L. frumenti* administration. **a**, **b** Correlation heat maps for the metabolites in each tissue. The correlation coefficient (rho) is shown in red (positive) or blue (negative) (LF, *L. frumenti*). **c** Number of significantly different metabolite correlations between the *L. frumenti* and PBS groups. **d**, **e** Graphical representation of the significant metabolite correlations. Each ribbon indicates a significant correlation between or within each metabolite class and the ribbon thickness represents the number of significantly correlated metabolites. The metabolites were ordered according to metabolite class, as indicated by the colored bar around the circumference

Numerous positive and negative correlations among the metabolites in the serum and liver were identified (Fig. 2a and b). Interestingly, opposing effects of *L. frumenti* administration on metabolite correlations in the serum and liver were observed (Fig. 2c). Serum gained 21.8% correlations on *L. frumenti*, whereas liver lost 15.1% correlations on *L. frumenti,* indicating maintenance and/or reorganization of metabolism in blood and liver.

To further characterize the correlations among the metabolites, the significant positive and negative correlations were classified according to metabolite class (Fig. 2d and e). Comparative analysis of intra-tissue metabolite correlations showed that unclassified compounds and xenobiotics had the strongest correlations with other metabolites in the serum and liver, respectively. Notably, there were more correlations between serum amino acids and other metabolites in the *L. frumenti* group, whereas there were slightly fewer in the liver. These differences in the correlations among the metabolites between the groups imply that metabolism is differentially organized in the two groups of piglets.

### *L. frumenti* induces differences in lipid and amino acid metabolism in early-weaned piglets

**Fig. 3:**
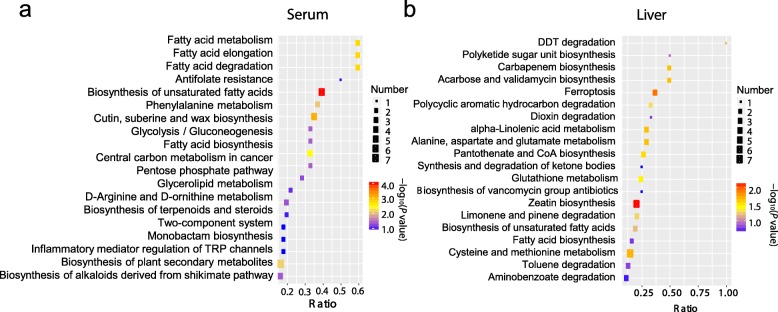
*L. frumenti* administration modulates amino acid and fatty acid concentrations in serum and liver. **a**, **b** KEGG pathway analysis of the altered metabolites in the serum and liver, respectively

In serum, the enriched pathways were mainly linked to circulating fatty acid metabolism and amino acid metabolism, especially the biosynthesis of unsaturated fatty acids (Fig. 3a). Pathway enrichment analysis showed that specific lipid classes were present at significantly different concentrations in the liver of *L. frumenti*-administration piglets, including those indicative of differences in alpha-linolenic acid metabolism, biosynthesis of unsaturated fatty acids, and fatty acid biosynthesis (Fig. 3b), such as long-chain fatty acids, polyunsaturated fatty acids (PUFA), phospholipids, and lysolipids. Metabolites implicated in amino acid metabolism, particularly in alanine, aspartate, and glutamate metabolism, and cysteine and methionine metabolism, were also highly affected by *L. frumenti*.

### Metabolites significantly associated with *L. frumenti* administration

**Fig. 4:**
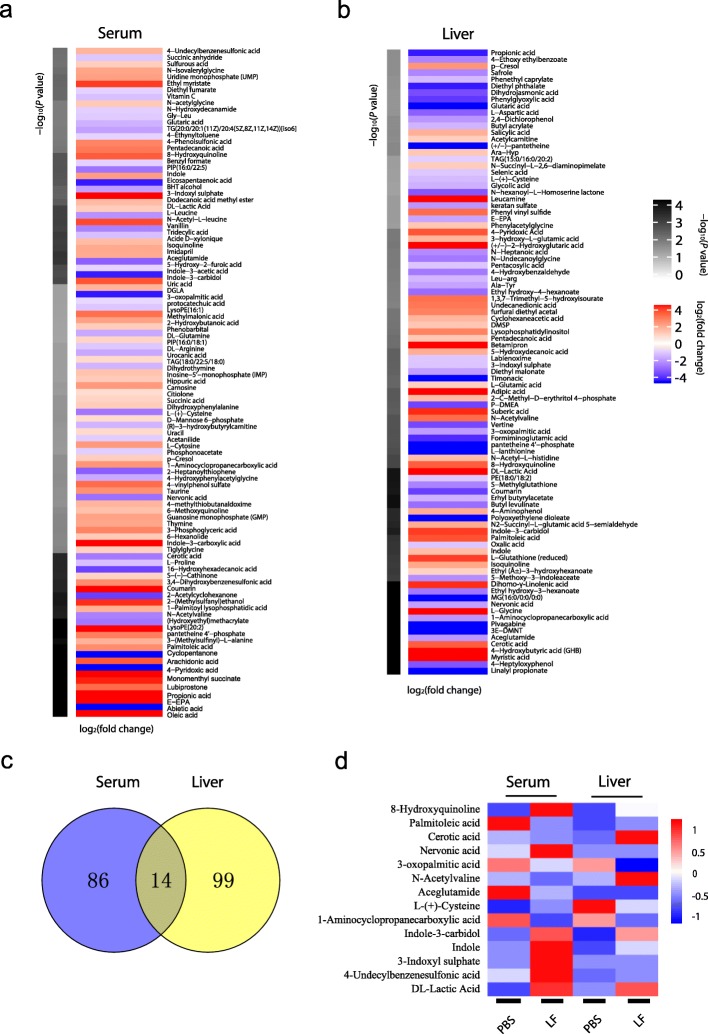
Metabolites significantly associated with *L. frumenti* administration. **a**, **b** Heat map of serum and liver metabolites affected by *L. frumenti* administration (LF, *L. frumenti*). **c** Metabolites affected in both serum and liver. **d** Relative amounts of similarly affected metabolites in serum and liver, transformed into Z scores in the heat map

To visualize the metabolites altered by *L. frumenti* administration, hierarchical clustering was used to categorize them on the basis of their differences in concentration. Among the altered metabolites, 59 metabolites were present at a higher concentration and 41 metabolites at a lower concentration in *L. frumenti*-treated piglet serum (Fig. 4a). Specific lipid classes were affected by *L. frumenti*, including long-chain fatty acids, poly-unsaturated fatty acids, phospholipids, and triacylglycerols. In the liver of the piglets, 55 metabolites were present at higher concentrations and 58 metabolites at lower concentrations (Fig. 4b). The concentrations of several amino acids and peptides were higher, whereas that of 3-oxo-hexadecanoic acid, an intermediate in fatty acid biosynthesis, was lower. Differences in the concentrations of long-chain fatty acids and poly-unsaturated fatty acids were also identified. Notably, 14 metabolites showed differences in concentrations in both the serum and liver (Fig. 4c), and Z scores were calculated to demonstrate these differences (Fig. 4d).

### *L. frumenti* administration affects nucleotide metabolism

**Fig. 5:**
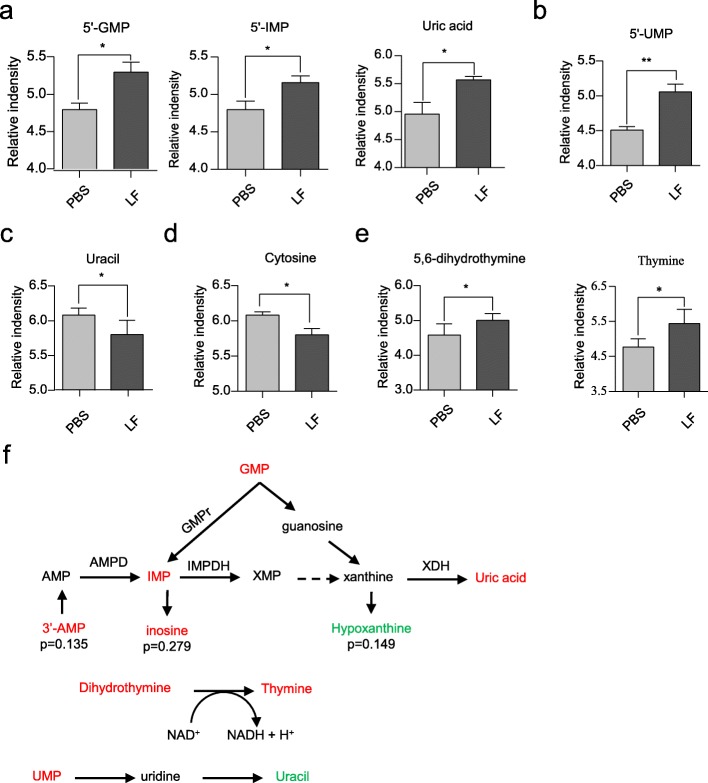
Regulation of nucleotide metabolism by *L. frumenti* in early-weaned piglets. **a** Purine nucleotide abundances in serum (LF, *L. frumenti*). **b**, **c** Uracil-related metabolite abundances in serum. **d**, **e** Cytosine and thymine-related metabolite abundances in serum. **f** Scheme showing how the metabolites of purine are interrelated and affected by *L. frumenti* administration. Metabolites significantly increased by *L. frumenti* are indicated by red text, while significantly reduced metabolites are green. Black metabolites were not measured. GMP, guanosine monophosphate; IMP, inosine monophosphate; UMP, uridine monophosphate. All values are shown as mean ± standard deviation (SD), and statistical significance was calculated using two-tailed Student’s *t*-tests. Differences were considered statistically significant at *P* < 0.05 (*), *P* < 0.01 (**). *n* = 5 piglets

The serum metabolomics also showed that GMP, IMP, and their metabolite uric acid were present at higher concentrations (Fig. 5a), indicating that *L. frumenti* may influence purine nucleotide metabolism in early-weaned piglets. Additionally, we found that uridine monophosphate (UMP) was also present at higher concentrations (Fig. 5b), while uracil (Fig. 5c) and cytosine (Fig. 5d) were present at lower concentrations. Another striking effect of *L. frumenti* on nucleotides was reflected in the presence of thymine and its precursor dihydrothymine at higher concentrations in treated piglets (Fig. 5e). The metabolic pathways of the nucleotides are summarized in Fig. 5f. In sum, our data suggest that *L. frumenti* promotes nucleotide catabolism in early-weaned piglets.

### *L. frumenti* administration promotes fatty acid oxidation and energy production in the liver of early-weaned piglets

**Fig. 6:**
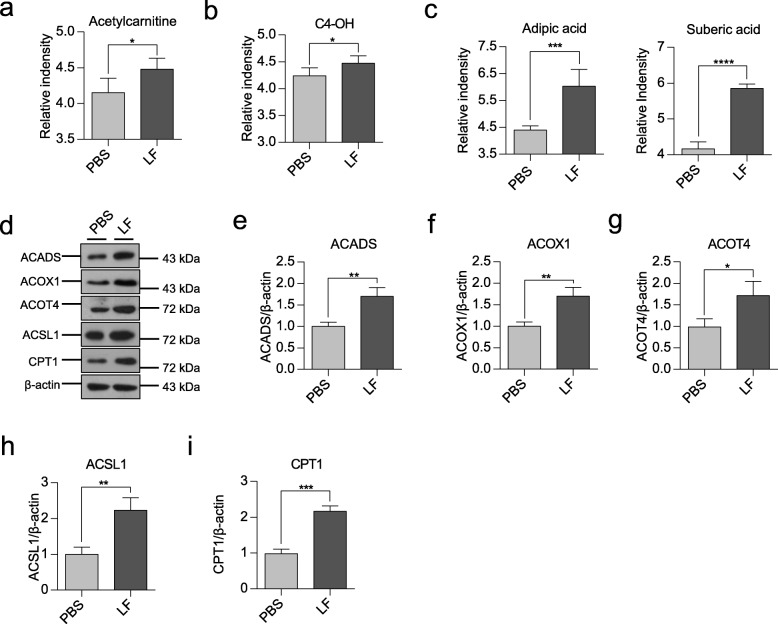
*L. frumenti* administration upregulates fatty acid β-oxidation in the liver of early-weaned piglets. **a**–**c** Fatty acid β-oxidation metabolites. **d** Metabolic enzymes involved in fatty acid β-oxidation. **e**–**i** Quantification of the proteins in (**d**). All values are shown as mean ± standard deviation (SD), and statistical significance was calculated using two-tailed Student’s *t*-tests. Differences were considered statistically significant at *P* < 0.05 (*), *P* < 0.01 (**),*P* < 0.001 (***), and *P* < 0.0001 (***). *n* = 5 piglets

Interestingly, previous studies have found that purine nucleotides are involved in energy conservation in the mitochondria of brown adipose tissue [16]. Therefore, we speculated that *L. frumenti* may regulate energy metabolism in early-weaned piglets. *L*-acetylcarnitine, which facilitates the movement of acetyl-CoA into mitochondria during the oxidation of fatty acids, was present at higher concentrations in the liver (Fig. 6a). Fatty acid β-oxidation metabolites, such as C4-OH, were also present at higher concentrations in serum of *L. frumenti* administration piglets (Fig. 6b). Dicarboxylate adipic acid and suberic acid, which are formed by peroxisomal β-oxidation, were present at higher concentrations in the liver of *L. frumenti* administration piglets (Fig. 6c), and we therefore speculated that the hepatic oxidation of fatty acids may be activated following *L. frumenti* administration. To test this possibility, the expression of key enzymes (ACADS, ACSL1, ACOX1, ACOT4 and CPT1) involved in fatty acid β-oxidation was measured (Fig. 6d-i). These five enzymes were significantly upregulated by *L. frumenti* administration. ACSL1 and CPT1 were significantly upregulated by *L. frumenti* administration. In addition, the protein ACOX1 and ACOT4 were upregulated. These results suggest that *L. frumenti* administration activates fatty acid β-oxidation in the liver of early-weaned piglets.

## Discussion

It is now widely accepted that gut microbial dysbiosis, induced by adverse dietary and environmental changes, is a key component of the etiology of post-weaning diarrhea and enteric infections. Studies conducted in recent years have identified the gut microbiota as a critical factor in gut health and metabolic homeostasis in early-weaned piglets [3]. *Lactobacilli* become established early in the intestine of piglets, and remain as one of the predominant bacterial communities throughout the lifetime of the pigs [17, 18], indicating that *Lactobacillus* may be a major factor in disease prevention and production performance [19, 20]. Our previous studies also indicated that oral administration of *L. frumenti* significantly increased the relative abundances of *L. frumenti* in early-weaned piglet fecals [12, 13]. In this study we found that oral administration of *L. frumenti* increased DL-lactic acid in serum and liver, suggesting that the administered *L. frumenti* may successfully colonize the gut. In accordance with our previous study [12], we observed that body weight was increased by oral administration of *L. frumenti*, indicating that *L. frumenti* may play an important role in early-weaned piglets. Growing evidence suggests that the gut microbiota regulate host energy metabolism. Our previous study demonstrated that *Lactobacillus gasseri* LA39 may act as a potential probiotic and stimulates energy production in early-weaned piglets [21]. The present study provides new insights into the effects of *L. frumenti* to increase the β-oxidation of fatty acid in the liver of early-weaned piglets.

In order to visualization of the correlations of intra-tissue metabolites altered by *L. frumenti*, we carried out correlation analysis. The results indicated clear differences between serum and liver. Increased correlations in serum may indicate that *L. frumenti* increased blood cycle and promote the metabolism in whole body, underscoring the role of *L. frumenti* in metabolic regulation. By contrast, the loss of correlations in liver implicates the reorganization of metabolism and may enhance liver functions. Interestingly, this observation may be the result of differences in the concentrations of unclassified compounds and xenobiotics, which are mainly absorbed from the gut and reach the liver through the hepatic portal vein.

Our data provide a resource to map the metabolic changes induced by *L. frumenti* gavage. *L. frumenti*-fed early-weaned piglets displayed significantly different serum and liver metabolic profiles from PBS-fed piglets, suggesting that *L. frumenti* affects whole-body metabolic regulation. Previous studies have shown that the gut microbiota can exert a substantial influence on host lipid metabolism [22, 23] and amino acid metabolism [24, 25]. Early weaning stress impairs development of mucosal barrier function in the porcine intestine, while our previous studies have proved that *L. frumenti* contributes to improve the intestinal epithelial barrier functions in early-weaned piglets [12]. Under these conditions, the liver may uptake more nutrients absorbed from the intestine and energy metabolism in the liver increases to metabolize these nutrients. The liver is a major site of fatty acid oxidation in animals [26]. Key enzymes involved in fatty acid β-oxidation,such as ACSL1, CPT1, ACOX1 and ACOT4, were upregulated in the liver of the early-weaned piglets with *L. frumenti* oral adminidtration. Our data indicated that *L frumenti* activated fatty acid β-oxidation to increase energy production in the liver of early-weaned piglets.

**Fig. 7:**
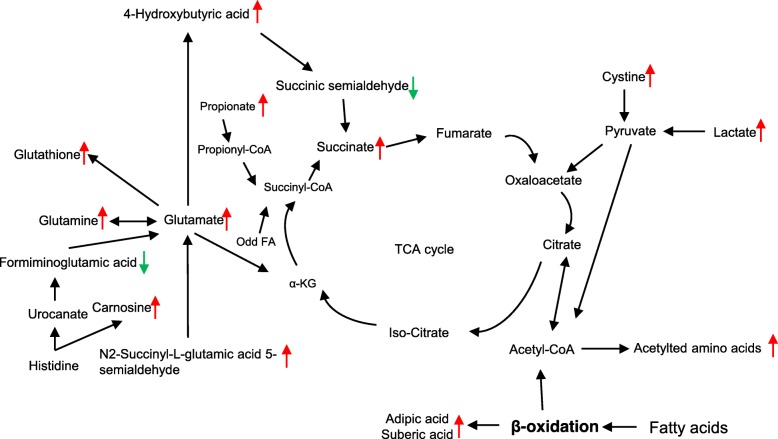
Effects of *L. frumenti* administration on lipid and amino acid signaling in the liver of early-weaned piglets. The affected metabolites are shown in bold. Red lines indicate upregulated and green lines indicate downregulated metabolites

In addition to β-oxidation byproducts, others metabolites that are involved in the tricarboxylic (TCA) cycle were also identified at different concentrations between PBS treatment and LF treatment (Fig. 7). Succinic acid is a vital intermediate in the TCA cycle, and in the present study serum succinic acid was present at significantly higher concentrations in *L. frumenti*-treated piglets. Furthermore, 4-hydroxybutyric acid can also be converted to succinic acid in liver [27]. Propionic acid is mainly derived from fermentation in the colon [28], and our data shows that *L. frumenti* maybe involved in upregulating fermentation in the colon, given the higher concentration of serum propionic acid detected. Propionic acid can be converted to propionyl-CoA, which enters the TCA cycle [29]. The lower concentration of propionic acid in the liver may indicate that propionic acid is utilized at a high rate in early-weaned piglets.

Suckling piglets have a special requirement for nucleotides, such as cytidine 5′-monophosphate (CMP), adenosine 5′-monophosphate (AMP), GMP, UMP, and IMP [30]. These nucleotides are involved in growth and maintenance, development, inflammation, and stress, and nucleotide demand increases during weaning. Significantly, restoration of intestinal mucosal integrity was observed in studies of nucleotide supplementation of weaning rats with diarrhea [31, 32]. Moreover, nucleotides cause diarrhea resistance in early-weaned piglets [33–35]. In addition, nucleotides, as energy donors and intermediates in biosynthetic and oxidative pathways, are involved in many cellular metabolic processes. Finally, dietary nucleotides are beneficial for ileal mitochondrial function in weaning rats with chronic diarrhea [36] and they have been reported to promote the growth and enzymatic maturation of the small intestine [37]. In the present study, increases in nucleotide concentrations induced by *L. frumenti* administration may have similar effects to direct nucleotide supplementation, with beneficial effects in early-weaned piglets, such as diarrhea resistance and improved intestinal epithelial barrier function, as observed previously [12]. IMP is produced by *de novo* synthesis and serves as a precursor for the formation of other nucleotides, such as GMP [38]. Moreover, nucleotides have also been shown to increase plasma immunoglobulin A (IgA) concentrations in early-weaned piglets [35], and this was consistent with our previous findings [12]. And we speculated that increased UMP or nucleotides maybe synthesized in gut by *L. frumenti*. While uracil decreases in serum, an increase expected, we hypothesis uracil maybe degrade to urea or used in β-alanine metabolism. Taken together, these results suggest that *L. frumenti* administration may benefit early-weaned piglets by increasing nucleotide concentrations.

The gut microbiota also regulates glutathione (GSH) and amino acid metabolism in the host [39]. Our KEGG analysis shows that *L. frumenti* regulates amino acid metabolism and glutathione metabolism in the liver of early-weaned piglets. GSH plays a key role in reducing oxidative stress, and most GSH synthesis occurs in the liver from cysteine, glutamate (Glu), and glycine (Gly) [40]. While lower hepatic Cys content may limit GSH production, an increase in hepatic Glu content may enhance the uptake of Cys from the blood via the cysteine-glutamate antiporter, resulting in a higher intracellular concentration of GSH. Fatty acid β-oxidation supplys energy for GSH synthesis. Amino acids also provide TCA cycle substrates. A previous study showed that serum glutamine (Gln) enters the TCA cycle to generate ATP in the liver of early-weaned piglets [41], after conversion to Glu and α-ketoglutarate (α-KG) [42].

Glu, Gln, and Gly are all essential precursors of proteins and nucleotides [43, 44], and a growing number of studies suggests that amino acids are important for the maintenance of gut development and health [45]. In the present study, *L. frumenti* administration was associated with higher concentrations of Glu, Gln, Gly, and Cys, which may all be involved in the maintenance of gut health and development in the early-weaned piglets [46–48]. By contrast, the lower serum concentrations of leucine (Leu) and proline (Pro) indicate that *L. frumenti* may result from protein synthesis in skeletal muscle, leading to an increase in amino acid absorption from the circulation. These results suggest that *L. frumenti* promotes gut health through amino acid regulation.

## Conclusions

In sum, oral administration of *L. frumenti* in early-weaned piglets affects metabolic homeostasis, especially lipid metabolism and amino acid metabolism, and induces fatty acid β-oxidation and amino acid use for energy production in the liver. *L. frumenti* administration also increases nucleotide concentrations, which may contribute to diarrhea resistance. In addition, *L. frumenti* may protect early-weaned piglets from oxidative stress by promoting GSH synthesis in the liver. These findings further highlight the importance of the gut microbiota for diarrhea resistance and demonstrate an effect of *L. frumenti* to promote energy production in early-weaned piglets.

## Data Availability

The datasets generated or analyzed during this study are presented in this manuscript and available to readers.

## Abbreviations

ACOT4: Acyl-CoA thioesterase 4
ACOX1: Acyl-coenzyme A oxidase 1
ACSL1: Long-chain fatty acid CoA ligase 1
C4-OH: 3-hydroxybutyrylcarnitine
CPT1: Carnitine palmitoyl transferase 1
GMP: Guanosine monophosphate
IMP: Inosine monophosphate
KEGG: Kyoto Encyclopedia of Genes and Genomes
PCA: Principal component analysis
UMP: Uridine monophosphate
